# Expression of Concern: Bridging the gaps in eating disorder care: a systematic and comparative review of guidelines for prevention, early intervention, and service delivery

**DOI:** 10.3389/fpsyg.2025.1729669

**Published:** 2025-10-31

**Authors:** Frontiers Editorial Office

With this notice, Frontiers states its awareness of serious allegations surrounding the article “*Bridging the gaps in eating disorder care: a systematic and comparative review of guidelines for prevention, early intervention, and service delivery*” published on 8th October 2025. Our Chief Editors, FCE and SCE, will direct an investigation in full accordance with our procedures. The situation will be updated as soon as the investigation is complete. UPDATE: This article has now been retracted. Please find the full retraction here: 10.3389/fpsyg.2026.1794398.

